# Supplementary figures and images for: Distribution of Antibiotic Resistance Genes in Kocuria Species
Source: Antibiotics (Basel). 2025 Oct 17;14(10):1041. doi: 10.3390/antibiotics14101041 (PMC12562059; doi:10.3390/antibiotics14101041)

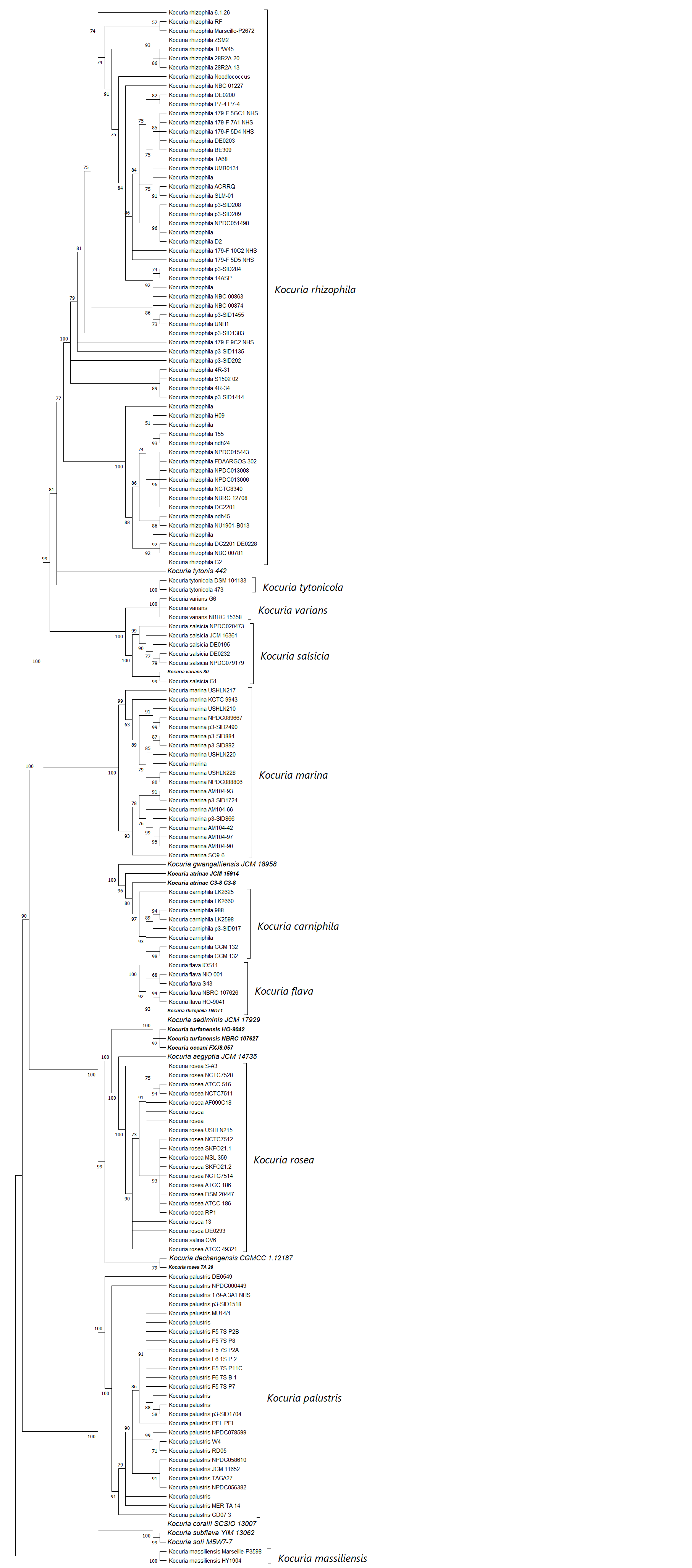

Supplement: Supplementary file 1 [file antibiotics-14-01041-s001.zip › Figure S1.tif]
